# Case Report: Optical coherence tomography to guide PCI of iatrogenic injury of the circumflex artery after minimally invasive mitral valve repair

**DOI:** 10.3389/fcvm.2023.1270541

**Published:** 2023-10-20

**Authors:** Dusan Borzanovic, Ivan Ilic, Dusan Nikolic, Ivan Stojanovic

**Affiliations:** ^1^ Department for Interventional Cardiology, Dedinje Cardiovascular Institute, Belgrade, Serbia; ^2^ Faculty of Medicine, University of Belgrade, Belgrade, Serbia; ^3^ Department for Interventional Cardiology, University Clinical Center Kragujevac, Kragujevac, Serbia; ^4^ Department for Cardiac Surgery, Dedinje Cardiovascular Institute, Belgrade, Serbia

**Keywords:** complication, mitral valve, circumflex, coronary, intervention, minimally invasive surgery, port-access, optical coherence tomography (OCT)

## Abstract

We present a case of a 42-year-old man who suffered an iatrogenic injury to his left circumflex (Cx) coronary artery after mitral valve (MV) repair surgery. After the patient suffered from myocardial infarction without ST-segment elevation following minimally invasive MV surgery, we performed repeated coronary angiography and optical coherence tomography (OCT), which revealed severe coronary stenosis of the dominant Cx caused by intramural hematoma. In addition, we proceeded with percutaneous coronary intervention and stent implantation.

## History of presentation

A 42-year-old symptomatic male patient in New York Heart Association (NYHA) functional class 3 was admitted to our hospital for elective MV repair surgery. The patient had no anginal complaints at the time of admission.

## Past medical history

The patient reported no prior medical history.

## Investigations

A preoperative transthoracic echocardiography (TTE) examination showed severe mitral regurgitation due to myxomatous degeneration of leaflets and bileaflet prolapse. The patient had a reduced left ventricular ejection fraction (LVEF) of 45%, along with increased left ventricular end-systolic (45 mm), end-diastolic (60 mm), and left atrial (51 mm) diameters. Preoperative coronary angiography revealed normal coronary arteries with left dominance, so minimally invasive “port-access” MV surgery was planned for the patient.

## Management

**Figure 1:**
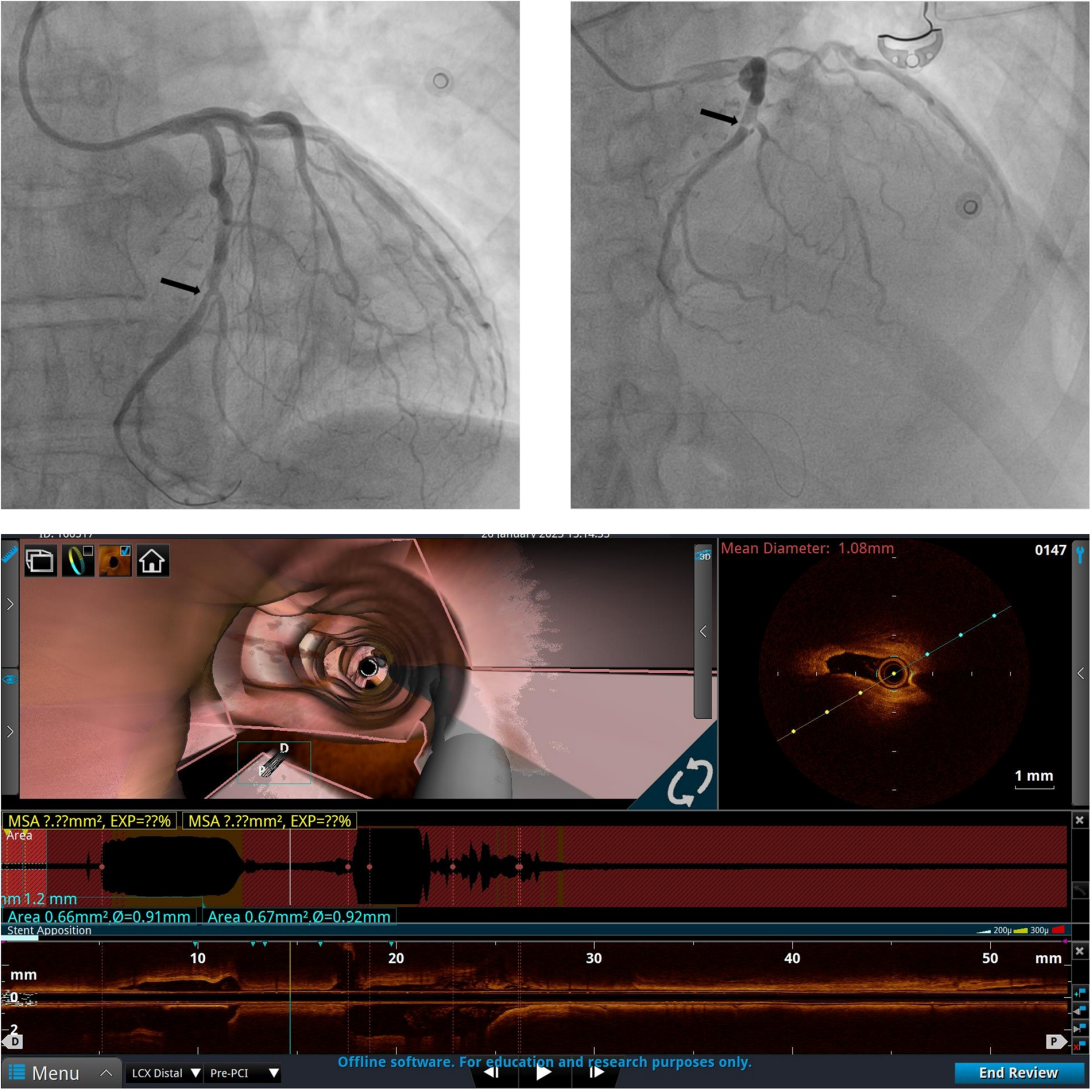
Left coronary artery angiogram and an OCT scan before PCI. The coronary angiogram with caudal and cranial projections of the left coronary artery along with the OCT image at the point of circumflex artery (Cx) critical stenosis are shown.

**Figure 2:**
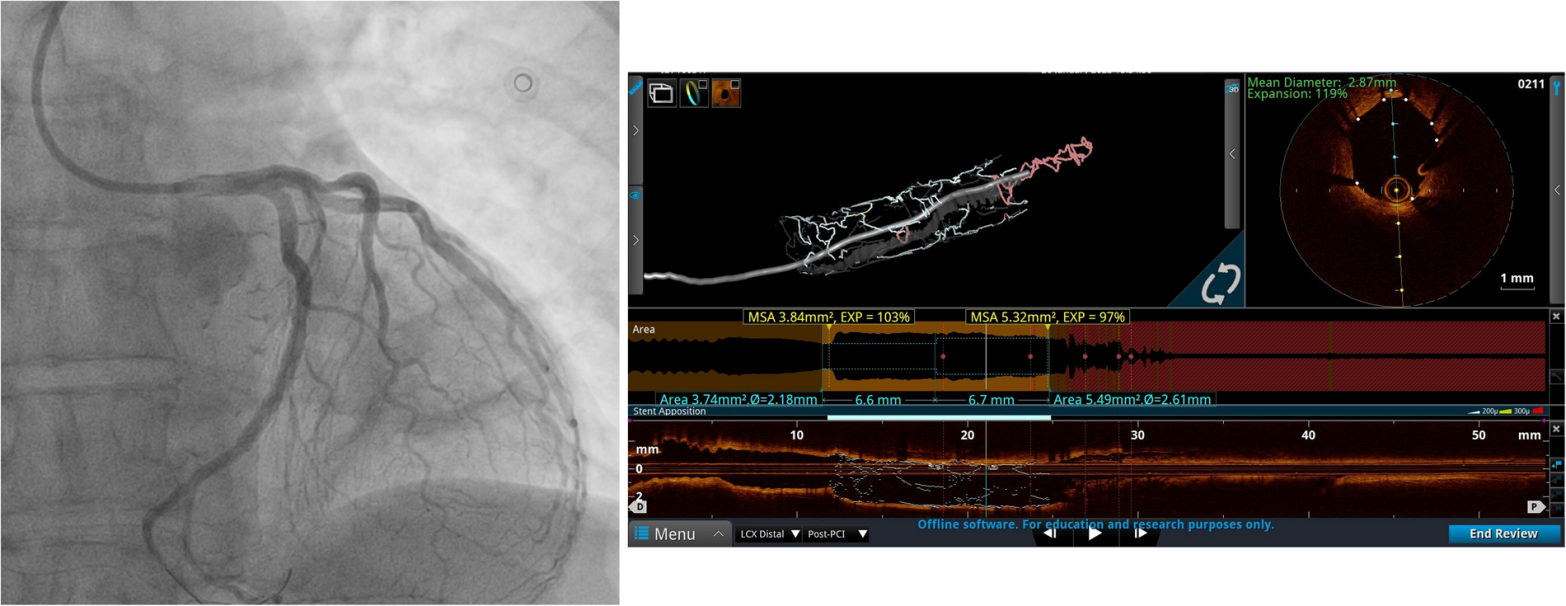
Left coronary artery angiogram and an OCT scan after PCI. The coronary angiogram in caudal projection after stent implantation and the OCT scan after PCI demonstrate good stent apposition and expansion.

The patient underwent “port-access” MV surgery through the fourth right intercostal space. Cardiopulmonary bypass was established via femoral access, along with the additional right jugular venous cannulation. Surgery revealed dominant P2 segment prolapse and a cleft at the A2–A3 junction. We performed the quadrangular resection of the prolapsing P2 segment, posterior leaflet sliding, and A2–A3 cleft closure with interrupted 5/0 polypropylene sutures. A 38-mm Seguin Semi-Rigid Annuloplasty Ring (St. Jude Medical, Saint Paul, MN, USA) was implanted afterward. An intraoperative transesophageal echocardiography (TEE) examination revealed fully competent MV, with a 9-mm coaptation line and preserved left ventricular contractility. Cardiopulmonary bypass weaning was uneventful, with minimal inotropic support. The patient was extubated the following morning, and the ECG monitoring pattern soon afterward changed to the third-degree AV block accompanied by episodes of nonsustained ventricular tachycardia. The patient did not report any chest pain. There was an increase in high-sensitive (hs) troponin value up to 51,827.1 pg/mL (upper reference normal value of 19.8 pg/mL). Repeated TTE exams in the intensive care unit (ICU) revealed new left ventricular posterior and lateral wall akinesia with LVEF decreasing to 40%. Pacing was not used given that the heart rate was around 50/min and that it responded to an increase in the doses of inotropic drugs with acceptable hemodynamics. Since there was a reasonable concern that the AV block was of ischemic origin, we decided to rush the patient to repeated coronary angiography and re-evaluate the need for permanent pacing after the procedure. Therefore, we decided to repeat coronary angiography, which revealed critical stenosis of the distal segment of the dominant Cx artery right after the takeoff of the third obtuse marginal (OM) branch. The lesion appeared to be caused by an inadvertent lesion of Cx by mitral valve annular sliding stitches (Figure 1). We decided to continue with percutaneous coronary intervention (PCI) and to perform the optical coherence tomography (OCT) exam first to evaluate the lesion and safely proceed with the procedure. Severe lumen reduction was found with an intramural hematoma in the distal segment with no atherosclerosis (Figure 1, Supplementary Videos S1, S3). We were not able to visualize surgical material (suture) crossing or adjacent to the vessel lumen. After the successful sequential predilatation with 1.5-mm × 15-mm, 2.0-mm × 15-mm, and 3.0- mm × 12-mm noncompliant balloons, a 3.5-mm × 25-mm sirolimus-eluting stent was implanted at 12 atm. Repeated OCT showed optimal stent expansion and apposition without edge dissection (Figure 2, Supplementary Videos S2, S4). No conduction disturbances occurred during or after revascularization, so we attributed them to ischemic origin and deferred pacemaker implantation. The later postoperative course was uneventful without the episodes of chest pain, ECG signs of ischemia and arrhythmias, and gradual decrease of troponin levels. TTE prior to discharge showed a slightly decreased LVEF of 45%–50% with hypokinesia of the basal segment of the septum. During the PCI procedure, the patient was started on clopidogrel with a loading dose of 600 mg and continued with 75 mg per day and aspirin with a loading dose of 300 mg and further continued with 100 mg per day. Oral warfarin was started on the second postoperative day.

## Discussion

An iatrogenic injury of the Cx artery after mitral valve surgery occurs due to the relative proximity of the mid-to-distal segment of the artery to the anterolateral commissure of the mitral annulus. In addition, in Barlow's disease, it is sometimes difficult to anticipate the exact position of the Cx due to annular dilatation and hypermobility of the mitral valve leaflets, which poses an additional risk of coronary artery injury (1–3).

The relative scarcity of published data does not provide accurate information about the incidence of this complication (it ranges between 0.3% and 1.8%), which makes it difficult to form a coherent strategy for the timely diagnosis and treatment of this potentially fatal complication (4, 5). The risk of iatrogenic myocardial infarction associated with Cx injury is slightly higher after MV repair (2.2%) compared to isolated MV replacement (1.7%), and there is no significant reduction in the incidence during minimally invasive MV repair (3).

Virmani et al. found an association between injury and the spatial relationship between the left coronary artery and MV annulus, further stating that the risk of injury is higher in patients with left coronary dominance/codominance (average distance of Cx from the mitral annulus was 4.1 mm for left dominance, 5.5 mm for codominance, and 8.4 mm for right dominance) (5). On the other hand, Pessa et al. did not find this association, but their study included only two patients with left coronary dominance (6). Our case supports the association of left coronary dominance with a higher likelihood of Cx injury after MV repair (6). Kishimoto et al. divided patients with mitral regurgitation (MR) into four groups (degenerative MR, atrial function MR, ventricular function MR, and Barlow's disease) based on the spatial relationship between the MV annulus and Cx on cardiac CT. They defined the X point as the location where the smallest distance between Cx and the mitral annulus was observed. Measurement of the distance between the mitral annulus and the closest point on the wall of the LCX artery was quantified and termed as the minimal coronary annular distance (mCAD). The median value for mCAD was set at 4.2 mm, and a value shorter less than 3 mm indicated an increased risk of coronary injury during mitral valve surgery. Notably, patients with left coronary dominance and patients with Barlow’s disease had shorter mCAD values (7). Unfortunately, we did not perform CT prior to surgery, which helped identify a patient at risk of Cx injury after MV surgery.

The mechanisms of Cx injury are multiple and include direct laceration or obliteration of the artery that can further lead to bleeding or thrombotic occlusion, distortion of the artery due to tissue retraction during extensive quadrangular resection, external compression, and kinking of the artery by the annuloplasty ring and the appearance of thrombus or hematoma in the blood vessel wall. Less common and potentially reversible mechanisms include air embolism and coronary vasospasm. In addition, severe calcification beyond the valve leaflets, anomalous origin of the Cx, extensive myxomatous valve degeneration, and the use of a large annuloplasty ring greater than or equal to 30 mm can contribute to the increased risk of Cx injury (8–10). In our case, the injury was most likely caused by a laceration of Cx resulting in the formation of an intramural hematoma verified on OCT recording rather than suture obliteration or needle puncture.

Although the treatment includes the urgent establishment of coronary blood flow, there is no clearly defined modality—the decision for percutaneous or surgical myocardial revascularization usually depends on the circumstances. If the LCX injury was diagnosed intraoperatively, it is recommended to perform urgent surgical myocardial revascularization (8–10), but if it was diagnosed postoperatively, the first choice is PCI due to its fast performance and avoidance of repeated sternotomy (7–10). However, surgical treatment should be considered in case of technical difficulties during PCI (difficult passage of the lesion, incomplete balloon expansion, absence of flow establishment) (11).

We used OCT to define the mechanism of Cx injury in our patients, and, to the best of our knowledge, it was the first case report that used OCT to elucidate the mechanism of Cx injury and guide intervention. Furthermore, by using 3D reconstruction, we were able to clearly see how the injury to the artery occurred and be confident that PCI would be an excellent treatment option. There have been cases where intravascular ultrasound was used in this group of patients, and we sincerely think that imaging should be mandatory in these patients prior to deciding optimal treatment options (12, 13).

## Discharge

The patient was discharged on the eighth day after the surgery with recommended triple therapy consisting of warfarin titrated to an international normalized ratio (INR) of 2.0–2.5, 100 mg aspirin, and 75 mg clopidogrel per day.

## Follow-up

At the 1-month visit, the patient was in stable condition without any complaints or signs of bleeding. On the next visit, it was decided to stop oral warfarin after 3 months and recommended to continue dual antiplatelet therapy (DAPT) for 12 months after the PCI procedure.

## Conclusion

OCT provides a valuable tool in determining the mechanism of Cx injury after minimally invasive mitral valve repair, helps to determine optimal treatment, and can guide percutaneous revascularization.

## Learning objectives

This study aims to review the mechanism of Cx injury during mitral valve surgery; evaluate the treatment options for this complex group of patients; evaluate the importance of intravascular imaging, especially OCT, in determining the mechanism of Cx injury after minimally invasive mitral valve repair; and evaluate treatment options and guiding PCI.

## Data Availability

The original contributions presented in the study are included in the article/**Supplementary Material**, further inquiries can be directed to the corresponding author.

## Supplementary material

Supplementary Video S1 OCT pullback of Cx artery before PCI.

Supplementary Video S2 OCT pullback after Cx PCI with stent implantation.

Supplementary Video S3 3-D OCT reconstruction prior to CxPCI.

Supplementary Video S4 3-D OCT reconstruction after CxPCI.
